# Thienothiopyransulfonamides as Complexing Agents for the Preparation of Dual Carbonic Anhydrase Inhibitors

**DOI:** 10.1155/MBD.1995.327

**Published:** 1995

**Authors:** Claudiu T. Supuran

**Affiliations:** 1 University of Florence Laboratory of Inorganic and Bioinorganic Chemistry Via Gino Capponi 7 Firenze 1-50121 Italy

## Abstract

Co(II); Zn(II) and Cu(II) complexes of two new sulfonamide carbonic anhydrase (CA) inhibitors, derivatives of thienothiopyran-2-sulfonamide, were prepared and characterized by analytic, spectroscopic, magnetic and conductimetric measurements. The new complexes are more potent CA inhibitors than the parent sulfonamides, with IC_50_ values around 0.1 nM, against isozyme CA II.


### THIENOTHIOPYRANSULFONAMIDES AS COMPLEXING AGENTS FOR THE PREPARATION OF DUAL CARBONIC ANHYDRASE INHIBITORS

Claudiu T. Supuran
University of Florence, Laboratory of Inorganic and Bioinorganic Chemistry,
Via Gino Capponi 7, I-50121, Firenze, Italy

**Abstract**: Co(II); Zn(II) and Cu(II) complexes of two new sulfonamide carbonic anhydrase (CA) inhibitors, derivatives of thienothiopyran-2-sulfonamide, were prepared and characterized by analytic, spectroscopic, magnetic and conductimetric measurements. The new complexes are more potent CA inhibitors than the parent sulfonamides, with $IC_{50}$ values around 0.1 nM, against isozyme CA II.

### Introduction

Heterocyclic sulfonamides were only recently investigated as complexing agents,[1,2] although some of them such as acetazolamide **1**, methazolamide **2** ,or ethoxzolamide **3** are widely used clinical agents[3] for the management of disorders associated with electrolyte secretion disequilibria.

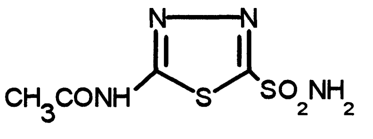

**1**

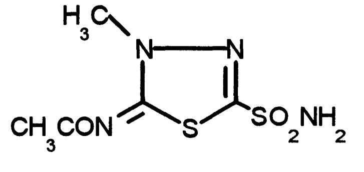

**2**

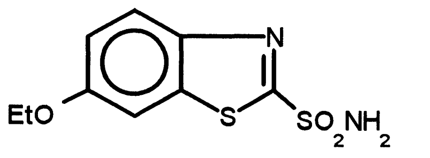

**3**

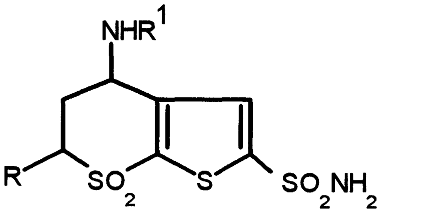

**4** a: R=H; $R^1$= $Me_2CHCH$

b: R=Me; $R^1$ = Et

It was only recently reported [4] that the metal complexes of sulfonamides **1-3** behave as very strong inhibitors of the zinc enzyme carbonic anhydrase (CA, EC 4.2.1.1). Their mechanism of action has also been explained by this group. [4,5] Thus, this type of CA inhibitors possesses a dual mechanism of inhibition, by means of sulfonamide anions and metal ions, formed by dissociation of the complexes in dilute solutions during the enzymatic assay.[4] The sulfonamide anions bind thereafter to the catalytically vital Zn(II) ion within the CA active site, whereas cations probably bind in the neighborhood of active site residue His-64 (which acts as a proton shuttle during the catalytic turnover [6]), disturbing in this way the whole catalytic cycle.[4,5] This dual mechanism also explains why metal complexes of heterocyclic sulfonamides are much stronger CA inhibitors as compared to the parent ligand sulfonamide.[1,4,5]

-------------

*Presented at EUROBIC II, Florence, Italy, August 1994.

Recently, Merck [7] developed a novel class of water soluble CA inhibitors, derivatives of thienothiopyran-2-sulfonamide, of type **4**, which are topically active antiglaucoma agents. [8,9] In this paper we report the first complexation study with this class of pharmacologically important compounds, and on the other hand, this is the first example in which a sulfonamide which does not possess endocyclic nitrogen atoms as putative donor atoms, is used for preparing complexes. Practically, the two sulfonamides used for the preparation of complexes are sezolamide **4a**, HSZA ((*R*)-5,6-dihydro-4-(2-methylpropyl)amino-4*H*-thieno[2,3-*b*]thiopyran-2-sulfonamide 7,7-dioxide) and dorzolamide **4b**, HDZA (5,6-dihydro-(*S*)-4-ethylamino-(*S*)-6-methyl-4*H*-thieno[2,3-*b*]thiopyran-2-sulfonamide 7,7-dioxide (both as hydrochlorides). Both ligands were used as pure enantiomers: (*R*) in the case of **4a**, and (*S,S*) in the case of **4b**, which contains two chiral centers. These are the enatiomers possessing the most potent CA inhibitory properties in this class of compounds. [7,9] The actual drug is **4b**, dorzolamide, recently introduced in clinical medicine in USA, whereas **4a** was the first compound from this class developed by Merck and abandoned thereafter for **4b**, which, containing a supplementary chiral center, presumably interacts more specifically with the enzyme. [8-10]

### Materials and Methods

Melting points were recorded on a heating plate microscope and are not corrected. FTIR spectra were obtained on thin films of pure compound, with a Perkin Elmer 1600 instrument, in the range 400 - 4000 $cm^{-1}$. Electronic spectra were obtained by the diffuse reflectance technique in MgO as reference, with a Perkin Elmer Lambda 17 apparatus. Conductimetric measurements were done in DMF solutions, at 25°C (concentrations of 1 mM of complex) with a Fisher conductimeter. Magnetic susceptibility measurements were done at room temperature by Faraday's method, using $CoHg(NCS)_4$ as standard. Elemental analyses were done by combustion for C,H,N with an automated Carlo Erba analyzer, and gravimetrically for the metal ions, and were ± 0.4% of the theoretical values.

Sulfonamides **4a,b** used in the syntheses were prepared as described in the literature. [7] Metal salts, organic reagents used for preparing the ligands **4** and solvents were from Aldrich and were used without additional purification. Bovine CA II was from Sigma Chemical Co. Inhibitors were assayed by Maren's micromethod [10], in the conditions of the E-I (enzyme-inhibitor) technique, at 0°C in veronal buffer. $IC_{50}$ values represent the molarity of inhibitor producing a 50% decrease of CA specific activity for the $CO_2$ hydration reaction.

### Synthesis of coordination compounds 5-10

An amount of 10 mMoles of sulfonamides **4a,b** as hydrochloride was dissolved in a solution obtained from 20 mMoles of NaOH and 15 mL water. The sulfonamide sodium salt obtained in this way was treated with a solution of metal salt ($MCl_2 .xH_2O$, where M = Co(II); Zn(II); and Cu(II) ), at molar ratio sulfonamide : M(II) of 2:1. The complexes **5-10** precipitated immediately, were filtered and air dried. Yields were very good (85-95%). Presumably, in these conditions the chirality of centers present in the thiopyran moiety of ligands **4** is not affected, as suggested by experimental data of ref. [7b]

### Results and Discussion

The new derivatives prepared in this study, containing sezolamide **4a** and dorzolamide **4b** as ligands and the following metal ions, Co(II); Zn(II) and Cu(II), of type **5-10**, are shown in Table I, together with their elemental analysis data (within ± 0.4% of the theoretical values).

The prepared derivatives were further characterized by spectroscopic, magnetic and conductimetric measurements. In Table II some of these data are shown, more specifically the sulfonamido vibrations in the IR spectra of the ligands **4** and complexes **5-10**, the electronic transitions in the diffuse reflectance spectra of the complexes as well as magnetic and conductimetric data of these compounds.

The IR spectra of complexes **5-10** differ little from those of the original legends, except for the two intense sulfonamide bands, which were shifted for the complexes with about 20 $cm^{-1}$ towards lower wavenumbers as compared to the corresponding bands of the ligands. This behavior is well documented for other complexes of heterocyclic sulfonamides, such as **1-3**, previously reported, [1,2,4,11,12] and clearly indicates that the deprotonated sulfonamido moiety interacts with the metal ions, a fact confirmed by X-ray crystallographic studies of several complexes containing acetazolamide **1**, methazolamide **2** or other sulfonamides of this type. [2,4c,11]

Table I: Complexes **5-10** prepared and their elemental analysis data.

| No. | Compound | Color | Analysis (calc./found) %M[a] | %C[b] | %H[b] | %N[b] |
|---|---|---|---|---|---|---|
| **5** | $[Co(SZA)_2]$ | violet | 8.0/7.9 | 35.9/36.1 | 4.6/4.7 | 7.6/7.5 |
| **6** | $[Zn(SZA)_2]$ | white | 8.8/8.5 | 35.6/35.5 | 4.6/4.4 | 7.5/7.5 |
| **7** | $[Cu(SZA)_2(OH_2)_2]$ | emerald | 8.2/8.0 | 34.1/34.1 | 4.9/4.7 | 7.2/6.9 |
| **8** | $[Co(DZA)_2]$ | violet | 8.3/8.0 | 34.0/33.8 | 4.2/3.9 | 7.9/7.8 |
| **9** | $[Zn(DZA)_2]$ | white | 9.1/9.2 | 33.7/33.5 | 4.2/4.0 | 7.8/7.5 |
| **10** | $[Cu(DZA)_2(OH_2)_2]$ | blue | 8.5/8.2 | 32.1/32.2 | 4.5/4.1 | 7.5/7.2 |

[a]By gravimetry; [b]By combustion

In the diffuse reflectance spectra of the Co(II) complexes **5** and **8**, an intense band with three maxima, around 16,250; 18,300, and 19,870 $cm^{-1}$, respectively was evidenced, which is characteristic for this ion in (distorted) tetrahedral geometry.[11-15] On the other hand, the magnetic moment of the Co(II) derivatives, around 4.3 BM, is characteristic for this geometry of Co(II). [12-16]For the two Cu(II) complexes, **7** and **10**, a structureless wide band centered at 16,200 $cm^{-1}$ was observed, similar to that of the acetazolamide complexes of Cu(II) previously reported by us [4a] and by Borras' group.[11] Correlated with a magnetic moment of 2.2 BM at room temperature, this is typical of Cu(II) in octahedral surrounding. [11]

Table II: Spectroscopic, magnetic and conductimetric data for complexes **5-10** as compared to ligands **4**.

| **Comp.** | **IR Spectra** [a], $cm^{-1}$ $\nu(SO_2)^s$ | $\nu(SO_2)^{as}$ | **Electronic Spectra** [b], $\nu$ ($cm^{-1}$) | $\mu_{eff}$ [c] (BM) | **Conductimetry** [d], $\Lambda_M$ ($\Omega^{-1}$ x$cm^2$x$mol^{-1}$) |
|---|---|---|---|---|---|
| **4a** | 1162 | 1368 | - | - | 114 |
| **5** | 1141 | 1347 | 16,245; 18,300; 19,870 | 4.35 | 24 |
| **6** | 1139 | 1348 | - | - | 28 |
| **7** | 1138 | 1346 | 16,200 | 2.21 | 19 |
| **4b** | 1159 | 1345 | - | - | 109 |
| **8** | 1140 | 1327 | 16,250; 18,300; 19,860 | 4.31 | 34 |
| **9** | 1139 | 1327 | - | - | 27 |
| **10** | 1139 | 1326 | 16,200 | 2.23 | 35 |

[a] FTIR spectra of thin films of pure compound; [b] Diffuse reflectance spectra in MgO as reference; [c] At room temperature by Faraday's method; [d] Solution $10^{-3}$ M, in DMF, at 25°C

From the conductimetric data of Table II one can see that the ligands are 1:1 electrolytes due to the fact that they are hydrochlorides. The same behavior was also observed for the corresponding sodium salts (data not shown), in contrast to complexes **5-10**, which are non-electrolytes. An interesting observation was that these complexes possess a larger water solubility as compared to similar compounds, containing sulfonamides **1-3** as ligands, previously reported. [2,4,17]

From the above data one can conclude that the new sulfonamides **4** used for the preparation of complexes in this study behave as bidentate ligands, similarly with the "classical" ligands of this type, acetazolamide **1**, methazolamide **2** and ethoxzolamide **3**. But in contrast to these sulfonamides, derivatives **4** do not possess endocyclic nitrogen atoms. Thus, their donor system is constituted by the sulfonamide (negatively charged) nitrogen, and the endocyclic sulfur atom. In this way five-membered chelate rings are formed with the complexed metal ions. Mention should be made that only in a unique other case such a donor system was previously evidenced for complexes of heterocyclic sulfonamides, i.e., in the dinuclear Pt(II) complex of ethoxzolamide **3**. [18] In all other cases, the endocyclic nitrogens were preferred as compared to the sulfur atoms for formation of chelate rings. [2]

The geometry of central ions in the prepared complexes is supposedly tetrahedral for the Zn(II) derivatives, pseudotetrahedral for the Co(II), and octahedral (with two coordinated water molecules) for the

Cu(II) derivatives (mention should be made that the two water molecules are lost at temperatures over 170°C (data not shown), supporting this statement).

Complexes **5-10** prepared in this study were tested for their ability to inhibit carbonic anhydrase (bovine isozyme II was used in these assays). In Table III $IC_{50}$ values are presented for these compounds, comparatively with the corresponding data of the free ligands **4a,b**.

Table III: CA II inhibition data with compounds **4-10**, determined by Maren's method. [10]

| Inhibitor | **4a** | **4b** | **5** | **6** | **7** | **8** | **9** | **10** |
|---|---|---|---|---|---|---|---|---|
| $IC_{50}$ (nM) | 0.60 | 0.25 | 0.15 | 0.18 | 0.09 | 0.10 | 0.12 | 0.07 |

It can be seen that all these compounds act as very potent CA II inhibitors. The parent ligands are active in nanomolar concentrations, but due to their dual mechanism of action, the complexes of sezolamide and dorzolamide are among the most potent CA inhibitors ever reported. In the series of the prepared derivatives, the Cu(II) complexes of both ligands are the most potent inhibitors, followed by the Co(II) and Zn(II) derivatives. Probably this can be correlated with the affinity of the corresponding cation to the CA II proton shuttle, which is the active site residue His-64. [4,5]

In conclusion, this is the first report of coordination compounds of two new sulfonamide antiglaucoma agents, recently introduced in clinical use. In addition to contributing to the study of coordination chemistry of such derivatives, the present study also highlights the putative use of these complexes for developing novel types of pharmacological agents containing biologically relevant metal ions.

### References

1. C.T.Supuran, in "Carbonic Anhydrase and Modulation of Physiologic and Pathologic Processes in the Organism", I.Puscas ed., Helicon, Timisoara 1994, pp. 29-111.
2. G.Alzuet, S.Ferrer, J.Borras and C.T.Supuran, *Roum.Chem.Quart.Rev.*, **1994**, *2*, 283-300.
3. a) T.H.Maren, Physiol.Rev., 1967, 47, 595-781; b) T.H.Maren, *Drug Dev.Res.*, **1987**, *10*, 255-276.
4. a) C.T.Supuran, M.Andruh, and I.Puscas, *Rev.Roum.Chim.*, **1990**, *35*, 393-398; b) C.T.Supuran, G.Manole and M.Andruh, *J.Inorg.Biochem.*, **1993**, *49*, 97-104: c) L. Sumalan, J.Casanova, G.Alzuet, J.Borras, A.Castineiras and C.T.Supuran, *J.Inorg.Biochem.*, *in press.*
5. C.Luca, M.Barboiu and C.T.Supuran, *Rev.Roum.Chim.*, **1991**, *36*, 1169-1173.
6. D.N.Silverman and S.Lindskog, *Acc.Chem.Res.*, **1988**, *21*, 30-36.
7. a) J.J.Baldwin, G.S.Ponticello, P.S.Anderson, M.E.Christy, M.A.Murcko, W.C.Randall, H.Schwam, M.F.Sugrue, J.P.Springer, P.Gautheron, J.Grove, P.Mallorga, M.P.Viader, B.M.McKeever and M.A.Navia, *J.Med.Chem.*, **1989**, *32*, 2510-2513; b) T.J.Blacklock, P.Sohar, J.W.Butcher and E.J.J.Grabowski, *J.Org.Chem.*, **1993**, *58*, 1672-1679.
8. M.F.Sugrue, P.Gautheron, J.Grove, P.Mallorga, M.P.Viader, H.Schwam, J.J.Baldwin, M.E.Christy and G.S.Ponticello, *J.Ocul.Pharmacol.*, **1990**, *19*, 233-238.
9. T.H.Maren, *J.Glaucoma*, **1995**, *4*,49-62.
10. T.H.Maren, *J.Pharmacol.Exp.Ther.*, **1960**, *130*, 26-29.
11. G. Alzuet, J.Casanova, J.Borras, J.A.Ramirez and O.Carugo, *J.Inorg.Biochem.*, **1995**, *57*, 219-234.
12. a) C.T.Supuran, *Rev.Roum.Chim.*, **1993**, *38*, 229-236; b) G.Manole, O.Maior and C.T.Supuran, *Rev.Roum.Chim.*, **1993**, *38*, 474-479.
13. C.T.Supuran, R.Olar, D.Marinescu and M.Brezeanu, *Roum.Chem.Quart.Rev.*, **1993**, *1*, 193-204.
14. I.Bertini, G.Canti, C.Luchinat and A.Scozzafava, *J.Am.Chem.Soc.*, **1978**, *100*, 4873-4877.
15. R.Han, A.Looney, K.McNeil, G.Parkin, A.L.Rheingold and B.S.Haggerty, *J.Inorg.Biochem.*, **1993**, *49*, 105-121.
16. E.S.Raper, *Coord.Chem.Rev.*, **1994**, *129*, 91-156.
17. C.T.Supuran and G.L.Almajan, *Main Group Met.Chem.*, **1995**, *18*, 347-351.
18. M.Andruh, E.Cristurean, R.Stefan, and C.T.Supuran, *Rev.Roum.Chim.*, **1991**, *36*, 727-732.

**Received: September 26, 1995 - Accepted: October 26, 1995 - Received in revised camera-format: November 10, 1995**

